# Bitter Tonic Mixture for Improving the Appetite

**Published:** 1912-01

**Authors:** 


Bitter Tonic Mixture for Improving the Appetite.—Huchard is credited by *Bulletin General de Therapeutique* with the formulation of the following bitter tonic:

℞ Tincture of cinchona,
Tincture of calumba,
Tincture of gentian,
Tincture of rhubarb,
Tincture of nux vomica, of each............f℥iss.

M. et Sig.: Fifteen drops in a little water before meals.—*Monthly Cyclopedia and Medical Bulletin.*

